# Crying Evils against the Profession

**Published:** 1876

**Authors:** 


*Crying Evils Against the Profession.*—Several disgraceful evils are now, and have been for a long time, existing among and against the medical profession. Among? Yes. The more shame it is for us that any of them should originate among our members. One of these evils is the habit of direct and indirect advertising. Too many professional men take occasion to advertise their whereabouts and skill; and if they do not directly do so, the editors of the local papers announce for them all their little notable cases as a matter of news; often the doctor himself furnishes the particulars. Who could ask a better advertisement? If it is a grand operation he has performed, then a large space must be granted in the columns of the newspapers, with a handsome puff for the doctor. Sometimes these are paid for by the line, and sometimes they are inserted without the knowledge of the doctor; but if he does not make any effort to suppress its frequent repetition, then he becomes indirectly privy to such advertising. I know at this time of a doctor who was once president of a State medical society, and now stands high as a member of the same, who often has such notices of operations performed by him in some of the city papers, and in one paper has a card announcing his readiness to accept all cases, and those from a distance provided for with good board, nursing, etc. If the professional man is to stand or fall according to his merits, how can these proceedings be construed as honorable? Few of our profession that have not noted these professional advertisers and newspaper medical men, who still claim perfect regularity, will have any idea how far this irregularity is carried on. Certainly it is time that local medical societies were moving in the matter.

Another glaring evil is the fusion of regular and irregular medical colleges. Notably one in Michigan, where six professors of the regular school deliver lectures to the homœpathic class daily, in immediate connection with their own proper classes, and at the same time and hours of the day. Then at the close of the term these same professors must examine the students of both classes, and pronounce upon their qualifications for graduation, alike for regular and irregular students, while the homœpathic student is examined by two additional homœpathic professors. Thus it comes about that six-eighths of the teaching received by these irregular students is given by professors claiming to teach regular medicine, and six-eighths of the responsibility for the graduation of these irregulars rests upon the six professors who teach and examine them. The mere dodge of leaving their names off of all the diplomas, and having them signed only by the president, etc., of the University, is, to use a vulgar phrase, "too thin." If these men can teach homœpathic students, side by side with regulars, why shall not these same students, after graduation, csnsult with each other whenever convenience may require it? The one is no more inconsistent than the other. Again, the cry that had the faculty backed down and not held their places, the homœopathists would have cried out a great victory because the regulars were afraid to meet them in intellectual contests, is also "too thin." Have not the regular profession stood ready to openly contest any point the irregulars might bring forward? We have nothing to lose; for if they have any new or valuable principles or remedies we, as regulars, are at liberty to adopt the same, and make them known to the world for the good of our common humanity; for we do not practice by the use of secret nostrums or exclusive dogmas, nor deny the use of any remedy, or the application of any principle, that may be found valuable in combatting disease.

Another crying evil is that committed by religious newspapers in advertising quack medicines. With very few exceptions, the religious newspapers of our country take the advertisment of any and nearly all patent medicines, and laud them highly to the public, without having the least idea of the nature of these nostrums, or whether they will have the least tendency to accomplish what they claim. Is this honest? Do not the editors of such papers admit such advertisement solely for the money paid for so doing? They must live, it is true: but must they take the risk of ruining the health of their patrons in order to acquire a livelihood? What a handsome spectacle! They teach religious doctrines on one side, and recommend nostrums on the other that claim to accomplish things beyond the power of any known remedies to do; and the worst of it is, the editors are fully aware of this fact, and hence, it places them, as religious teachers of the public, in the attitude of giving Gospel truths in one column and a well set *lie* in another. Bread for the soul in one hand, poison for the body in the other. Happy spectacle! If these nostrums were all they are represented to be, would or could accomplish the great cures claimed for them, then there could be found some excuse for such papers taking them up and advertising them broad cast over the land; but every man, not overly credulous or a fool, must know when he reads them that they are well-gotten up lies to deceive the public into purchasing and using them, so that the nostrum vendor may fill his coffers with gold, at the expense of the lives and blood of their fellow-men, and the religious newspaper editors become their aiders and abettors in this criminal business; I can call it by no milder phrase, for having seen men hurried to their graves by some of these nostrums, it has been presented in the gravest light. The cry of these nostrum vendors that physicians descry them because they cut off their practice is false, for we know that the more patent medicines a community tries, the greater their demand for intelligent advice; and how very often it occurs that some one is taken ill with some dangerous disease that needs prompt and skillful management, but unaware of his great danger, resorts to some patent sugar-coated purgative pellets, or other nostrum, and awaits their expected happy removal of his physical ailments, only to find himself greatly deceived in his expectations, and then has to call on his family physician at last, from whom he learns, or his family learns, the great danger he is in, and how much valuable time has been lost that should have been used in well-directed efforts in combatting a serious disease. A short time of active practice will develop these cases in numbers beyond our expectation. Let the medical press bring its wholsome influence to bear against these evils with all its power, and though it may take much time to eradicate them, nevertheless its power for good will soon be felt, and great good to humanity will result therefrom. T. C. S.

*Kind Words.*—We are in receipt of many encouraging communications from medical brethren touching the character of our journal, for which we return thanks.

It would be pleasant to publish them all, if our space would permit. We make room in our present issue for two of these, which will be found below. We find in these the most gratifying assurances that our efforts in behalf of the medical literature of the South are beginning to be appreciated, and that the intelligent men of the profession are alive to the importance of building up and sustaining a first-class home journal. We do trust that every physician in the country will act upon the timely and well meant suggestions of esteemed correspondents in the letters referred to.

CRYSTAL SPRINGS, MISS., February 7, 1876.

*Dr. Thos. S. Powell*—Your favor of January 26, acknowledging the receipt of subscription money, to hand. I am glad it reached you in due time. I simply cannot get along without THE RECORD; I find it indispensable—all that is necessary, being a delightful companion; hope to be able soon to send you some new subscribers. Wishing you personal prosperity and continued success for the pet of the profession, THE RECORD, I am, sir, yours truly, T. P. LOCKWOOD.

